# Projection of August 2021 pumice dispersion from the Fukutoku-Oka-no-Ba eruption in the western North Pacific

**DOI:** 10.1038/s41598-023-31058-0

**Published:** 2023-03-09

**Authors:** Yu-Lin K. Chang, Iona M. McIntosh, Toru Miyama, Yasumasa Miyazawa

**Affiliations:** 1 Application Laboratory, Japan Agency for Marine-Earth Science and Technology, Yokohama, 236-0001 Japan; 2 Solid Earth Geochemistry Research Group, Japan Agency for Marine-Earth Science and Technology, Yokosuka, 237-0061 Japan

**Keywords:** Natural hazards, Ocean sciences, Solid Earth sciences

## Abstract

Marine hazards often occur unexpectedly. Long-term (> few weeks) projections are sometimes needed to predict the potential route of drifting targets (e.g. pumice, oil, shipwreck) in order to prevent further disaster, yet reliable long-term forecast data may be unavailable. The present study examined the long-term projection of pumice dispersion originating from the 2021 submarine eruption of Fukutoku-Oka-no-Ba volcano, Japan, based on hindcast reanalysis of the past 28 years of wind and ocean currents using the particle tracking method. The ensemble distribution showed a wide dispersion, which was dominated by the ocean currents. By contrast, wind provided a relatively uniform transport. Apart from the prevailing wind, typhoons also play a role in affecting pumice dispersion. The multi-year simulation provides a general view of pumice dispersion accounting for different uncertainty, which could be used for deducing the potential dispersion under different wind and ocean conditions.

The shallow submarine eruption in 2021 of Fukutoku-Oka-no-Ba volcano, located 60 km south of Ioto island in the northwest Pacific Ocean, created a mass of floating pumice rocks that eventually drifted over 1300 km to arrive in the Okinawa islands. This is the 5th major pumice raft event in the world since 2000, following rafts in the Tonga arc in 2001, 2006, 2019, and the Kermadec arc in 2012^1–4^. Of these recent events, the 2021 raft is particularly important for pumice raft studies because of the frequency and quality of satellite imagery covering both its eruption and subsequent dispersal as well as its diverse environmental and economic consequences. The eruption began at 05:57 (local time, JST) on 13th August, producing a sustained steam-rich subaerial plume reaching a maximum height of ~ 16 km above sea level before transitioning to intermittent Surtseyan eruptive activity that finally ceased by 16:00 on 15th August^5^. Multiple satellites imaged the formation of the pumice raft (e.g. Figure 1). Image analysis in Maeno et al.^6^ demonstrated that the pumice raft was seen accumulating on the sea surface above the submarine vent from 08:00 JST on 13th August, initially spreading radially around the vent and then dispersing towards the northwest until 12:00 JST, after which it was obscured from satellite view by the subaerial eruption plume. A subsequent pulse of eruptive activity on the afternoon of the 14th August may have produced a second mass of pumice in addition to the initial raft, but all raft pumice were confirmed to have left the vent area by 12:50 on the 15th, at which time the observed raft area was ~ 287 km^2^^7^.

**Figure 1:**
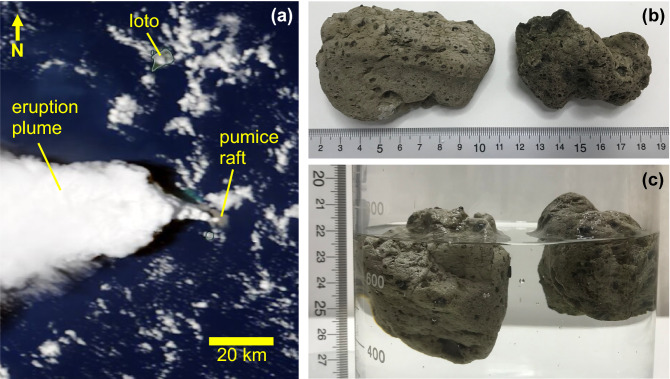
(**a**) Satellite image taken during the eruption on 13th August 2021. Pumice raft (grey mass) is accumulating on the sea surface above the submarine vent and beginning to disperse to the northwest. Image source: NASA Worldview, taken by Terra satellite during its descending/day orbit at 2021/08/13 00:50 UTC (09:50 JST). (**b**) Two pieces of raft pumice collected from Japan coastline (Ito port, Chiba) on 9th December 2021. Scale shows centimeters. (**c**) Same two pieces of raft pumice floating in water. The submerged portion is acted upon by ocean currents (draft) while the portion exposed above the waterline is acted upon by surface winds (windage). Scale shows centimeters.

Subaerial ash plumes can cause numerous hazards along the path of the plume during the eruption, including respiratory problems, damage to crops and infrastructure and closure of airspace to aviation, but after the eruption ends most ash rapidly falls out from the atmosphere. By contrast, the slow but sustained dispersal of floating pumice means that many of the hazardous and economic impacts of pumice rafts do not occur until weeks after the eruption when the raft reaches shipping lanes or coastlines. In Japan, the impacts of the 2021 pumice raft were numerous and long-lasting. Banks of pumice clogged Okinawa ports and disrupted fishery operations due to the damage that pumice can cause to boat propellors and engines (e.g. 206 boats reported pumice-induced engine trouble and 45 were temporarily disabled^8^,); concerns were also raised that power plants and other industries that use shallow seawater for cooling water could be damaged (e.g. https://www.asahi.com/ajw/articles/14473561). Large fish and turtles died after ingesting pumice pebbles mistaken for food and local ecosystems were also impacted by the ability of pumice rafts to block sunlight and affect air-sea exchange and other biological processes (e.g. https://english.kyodonews.net/news/2021/11/9a1c0bc5f4d4-areas-near-tokyo-await-arrival-of-floating-pumice-mass-damage-feared.html;^8^). Moreover, the tourism-reliant Okinawan economy was further damaged by the inundation by pumice of popular beaches, although this impact cannot be easily distinguished from the simultaneous drop in tourism during the Covid-19 pandemic. Ultimately, the economic impacts of the 2021 pumice raft in Okinawa have so far been estimated at more than 13 million USD (http://english.ryukyushimpo.jp/2022/02/22/34456/, https://www.pref.okinawa.jp/site/kankyo/seibi/documents/karuishitaisakukaigi_6_220215.pdf).

Following observation of a pumice raft or other floating hazard, scientists and local government are urged to obtain long-term (several weeks to months) predictions of dispersal direction and speed to enable potentially affected areas to be warned with sufficient time before the raft arrives to act to protect human life and perhaps mitigate damage to coastal infrastructure and ecology. The two major inputs for simulating pumice dispersal are wind and ocean currents. Wind and ocean current data, however, have limitations for providing long-term predictions. Uncertainty in future forcing and the potential for a slight perturbation leading to great divergence makes long-term forecasting a challenging task. Instead, a reliable forecast period may be only a few days (e.g., 2 days in the Gulf of Mexico^9^). Nevertheless, such short-term prediction may not be sufficient because decision making and taking action to mitigate potential disasters also takes time.

When long-term prediction is not available or possible, hindcast reanalysis of wind and ocean currents that cover different variability in past years can provide crucial insights. A similar method has been used to investigate the ensemble trajectories of the 2010 oil spill event in the Gulf of Mexico^10^. The present study aims to provide a long-term projection of pumice dispersal based on historical wind and ocean reanalysis from 1993 to 2020. The results are compared against satellite observations to examine the feasibility of ensemble analysis. Other alternative methods for long-term projection are also discussed.

## Materials and methods

The data-assimilative ocean circulation model that provided the three-dimensional currents and hydrological fields used for particle tracking in the present study is a modified version of the Japan Coastal Ocean Predictability Experiment 2 (JCOPE2M,^11^). The model domain of JCOPE2M encompasses the western North Pacific (WNP) (10.5–62°N and 108–180°E), with a horizontal resolution of 1/12° (8–9 km) and 46 vertical layers. The external forcing to drive JCOPE2M includes wind stresses and net heat/freshwater fluxes at the sea surface converted from the six-hourly atmospheric reanalysis produced by the National Centers for Environmental Prediction/National Center for Atmospheric Research (NCEP/NCAR). Satellite sea surface temperature from Himawari-8, sea surface height from Salto/Duacs altimeter products, and in situ temperature and salinity data from Global Temperature-Salinity Profile Program were assimilated into the model based on a multi-scale three-dimensional variational method^11^. JCOPE2M is available from 1993 to the present.

The wind vector data used for windage (a fraction of the wind vector, which accounts for the direct action of the wind on the floating pumice) in the particle tracking is from daily NCEP Reanalysis-2 based on NCEP/NCAR global reanalysis, which has a 2.5-degree horizontal resolution. The data are available from 1979 to 2021, and the period from 1993 to 2020 was used in the present study. For a sensitivity experiment, daily ECMWF-ERA5 reanalysis wind with higher 0.25 degree resolution were used for selected years of 2004 and 2021.

The passive particle-tracking method was used to investigate pumice dispersion after the eruption. The reanalysis data from JCOPE2M provided the background daily ocean current. The tracking scheme was based on the fourth-order Runge–Kutta method^12^ with a tracking time step of one hour. Vertical motion was not included in this study; instead, particles were tracked horizontally at the sea surface. Considering that part of the pumice was above the waterline and exposed to the air (Fig. 1c), windage was set to nonzero. A typical windage value of 1% (i.e. windage is 0.05 m/s eastward for 5 m/s westerly wind) based on previous studies^4,13^ as well as values of 2% and 0% (i.e. for considering no direct wind effect) were applied in the present study. The area for initial particle release was set as a 20 km by 20 km square around the eruption site (24.288°N, 141.482°E) in a 2 km horizontal interval. Particles were released at the sea surface on 13th, 14th, and 15th August (i.e., during the eruption period) each year, and tracked for ~ 140 days until 31st December when the amount of pumice arriving in the Okinawa Islands had significantly reduced compared to the amount arriving in November. Simulation periods covered from 1993 to 2020, with the 2021 eruption year excluded to fit the assumption of no long-term forecast data available when the sudden event occurred. Random-walk was not included in the particle-tracking scheme because of our emphasis on the ensemble methodology. Apart from the various windage conditions described above, additional experiments were also conducted to consider wind effect only (2% windage, no ocean currents), and geostrophic ocean current only (0% windage, with no surface wind effect on ocean current).

**Figure 2:**
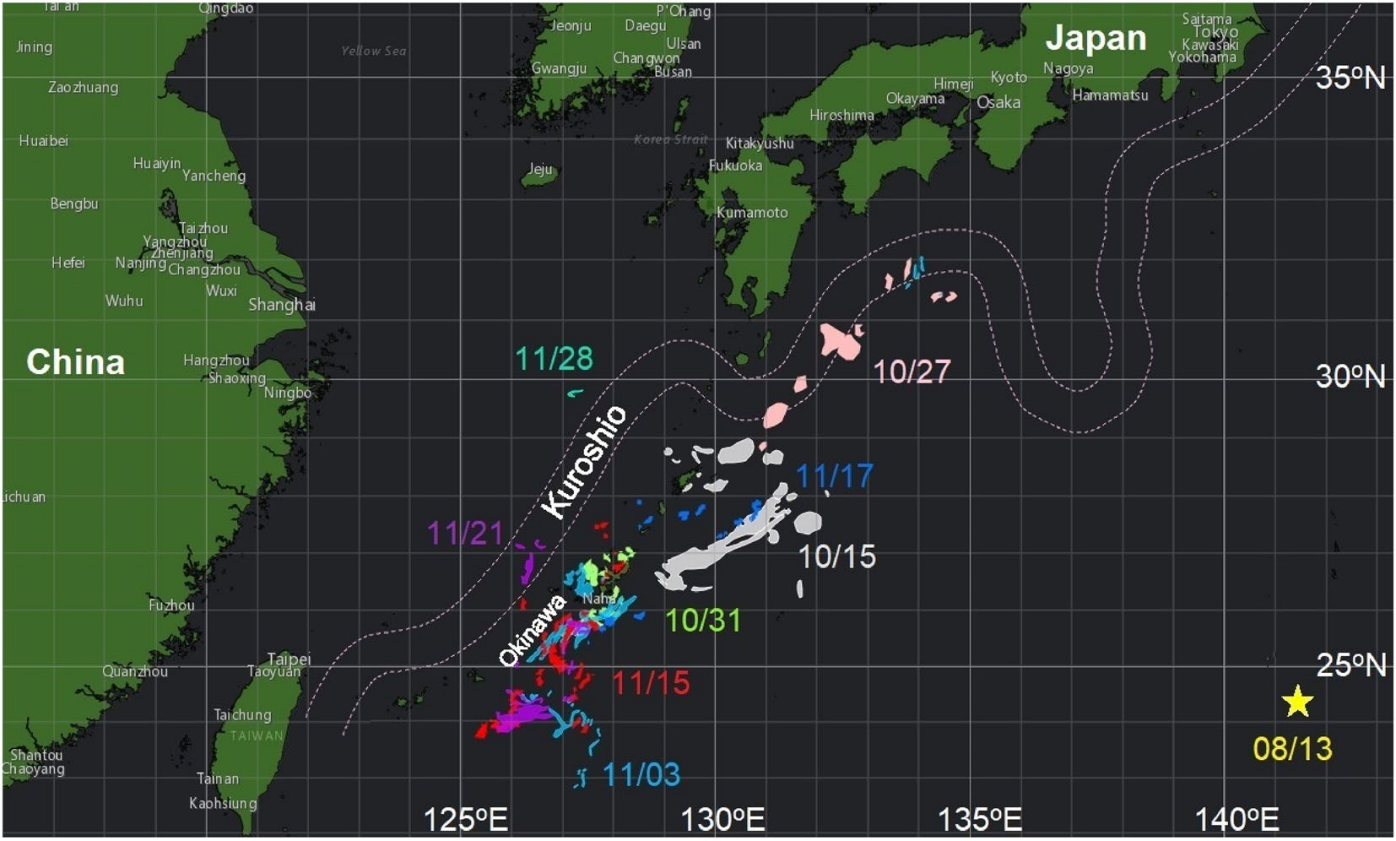
Satellite-observed pumice locations and dates (MM/DD). Different color shadings represent different observation dates; corresponding dates are marked in the same color. Yellow star shows the location of Fukutoku-Oka-no-Ba where the eruption began on 13th August. Continents are shaded dark green. Dashed contours show the mean location of the Kuroshio during the observation period. Pumice locations were observed by satellite GCOM-C (Shikisai) and Sentinel-2, and figure is generated using ArcGIS online system (https://www.esrij.com/products/arcgis-online/).

Pumice locations were observed by satellite GCOM-C (Shikisai) and Sentinel-2 based on the visible and infrared bands analyzed by Japan Aerospace Exploration Agency (JAXA, https://earth.jaxa.jp/karuishi/; Fig. 2). The normalized visitation frequency was calculated by the number of particles arriving at each grid point divided by the total number of released particles. The time-evolving distribution calculated the average time taken from the eruption site to each grid point.

## Results

**Figure 3:**
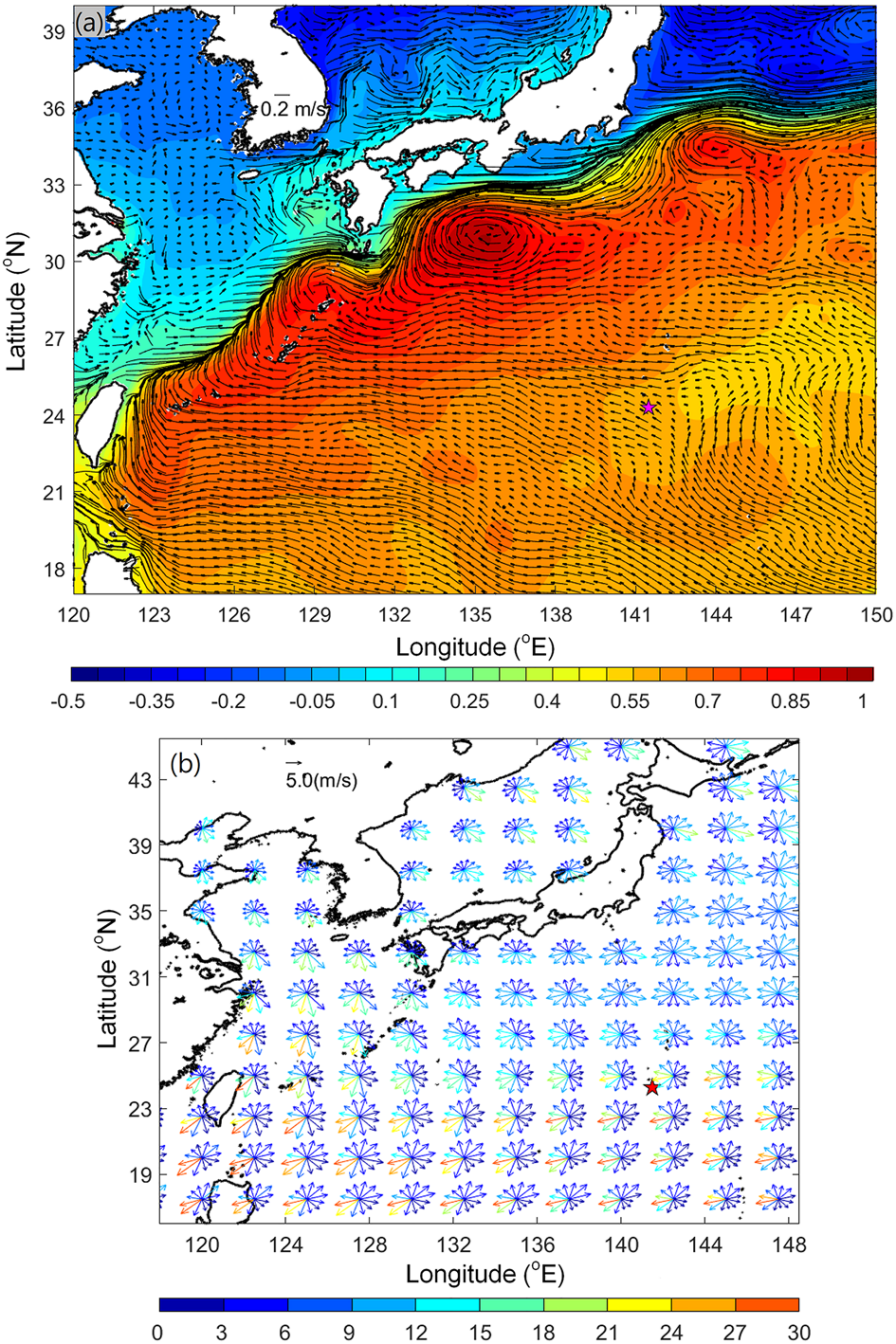
Ensemble-averaged data for the August to December period from 1993 to 2020 (**a**) Ocean surface currents (vectors, m/s) and sea surface height anomaly (SSHA, color, meter) (**b**) Wind roses in the western North Pacific, showing winds (vectors, m/s) and percentage occurrence (color) in indicated directions. Star indicates the eruption site at Fukutoku-Oka-no-Ba. Figure is created using MATLAB R2011b (http://www.mathworks.com/).

Satellite imagery captured the timing of pumice arrival near the Okinawa Islands, by which point the initial erupted mass of pumice had split into multiple smaller rafts (Fig. 2). Most pumice had reached 132°E by 15th October, nearly two months after the eruption. Some other pumice rafts arrived at Okinawa Island and the sea area south of Shikoku by early to mid-November. A great percentage of the pumice rafts were observed east of the Kuroshio, whereas only a few pumice rafts were found west of the Kuroshio in the East China Sea in late November. The eruption site is located inside the Kuroshio recirculation region where the mean current is westward (Fig. 3a,^14^). In the same region, mesoscale eddies generated by the Subtropical Countercurrent were often observed. The pumice rafts were carried westward by the mean current, and may also be trapped and transported by STCC eddies and arrived at Okinawa before being entrained into the Kuroshio (Fig. 3a).

Owing to a high percentage of gas-filled bubbles, pumice rock floats on the sea surface, with part of it underwater, and part of it exposed to the air (Fig. 1c). Accordingly, pumice is not only transported by ocean currents but also affected by the wind. A previous study suggested windage plays a key factor in pumice dispersal^4^. Applying a typical value of 1% windage to a wind speed of 5–10 m/s in the subtropical region will lead to 0.05–0.1 m/s speed contributed by wind, which is comparable with the surface current speeds in the Kuroshio recirculation region (Fig. 3a). The composite wind speed and direction between August and December from 1993 to 2020 in the western North Pacific is shown in Fig. 3b. The wind can blow in any direction in the western North Pacific. The prevailing winds were southwestward, westward, and northwestward between the eruption site and Okinawa, together accounting for more than 50% of total occurrence (Fig. 3b). Eastward and southward winds comprised a lower percentage of between 2–10% in each direction. Wind speed varied from 2 to 11 m/s, with a standard deviation of 1.8 m/s. Setting 1% windage, the wind-induced transport would then be 0.02 to 0.11 m/s, transporting towards Okinawa and Taiwan.

**Figure 4:**
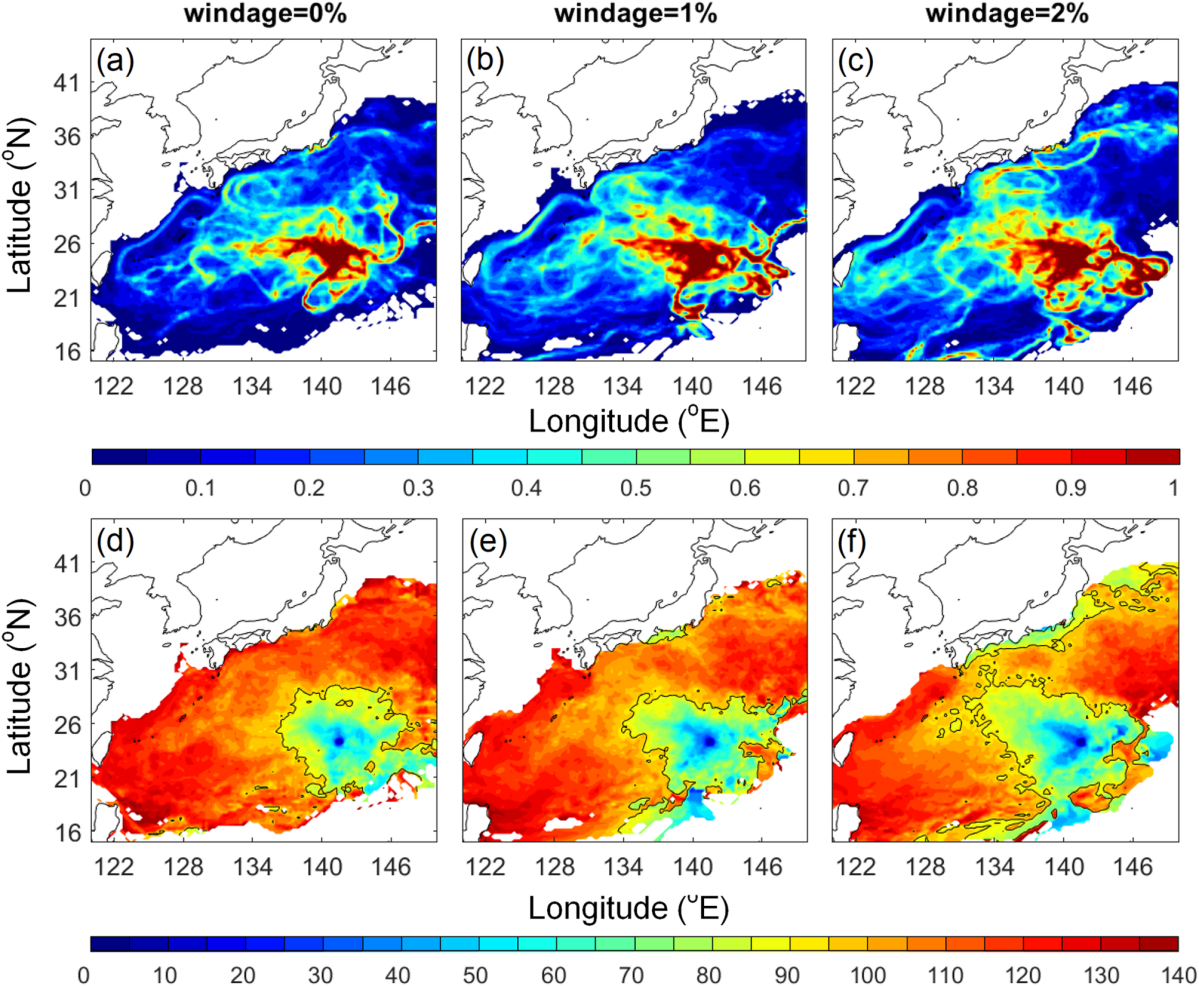
(**a**–**c**) Normalized visitation frequency and (**d**–**f**) time-evolving distribution (days) based on 28 years’ (1993–2020) daily hindcast ocean currents with windage of (**a**, **d**) 0%, (**b**, **e**) 1%, and (**c**, **f**) 2%. 90-day contour is shown in black in (**d**–**f**). Figure is created using MATLAB R2011b (http://www.mathworks.com/).

A composite of pumice dispersal based on 28 years of simulation showed a wide distribution of virtual pumice extending from 120 to 150°E, and from 15 to 40°N (Fig. 4). Note that the visitation frequency shown in Fig. 4 counts all particles in all simulation years. 0%, 1%, and 2% windage simulations showed a similar dispersal, yet the time-evolving distributions were different. 90 days after the eruption, pumice in 0% windage arrived around 136°E, 1% windage pumice reached 134°E, and westmost pumice in 2% windage appeared as far as 128°E near Okinawa. 2% windage seemed to provide a better fit for the 2021 eruption, as the actual pumice arrival to Okinawa was about 3 months (Fig. 2).

For a rough comparative estimation of windage using the time periods between the eruption and the pumice arrival to Okinawa as observed by satellite imaging (Fig. 2), the straight-line distance from the eruption site to the east of Okinawa where pumice was observed on 15th October was about 1100 km with a time interval of about 2 months, and the arrival to the west of Okinawa by the middle of November had a distance of 1500 km and period of 3 months. The straight-line pumice drifting speeds were therefore 0.192–0.212 m/s. The mean ocean current speed between the eruption site and Okinawa from August to November between 1993 to 2020 was 0.102 m/s, accounting for ~ 50% of this drifting speed. The mean wind speed in the same spatial domain and period was 5.20 m/s. Using these ocean current and wind speed values, the estimated windage for the 2021 dispersal based on straight-line drifting is thus 1.7–2.1%.

**Figure 5:**
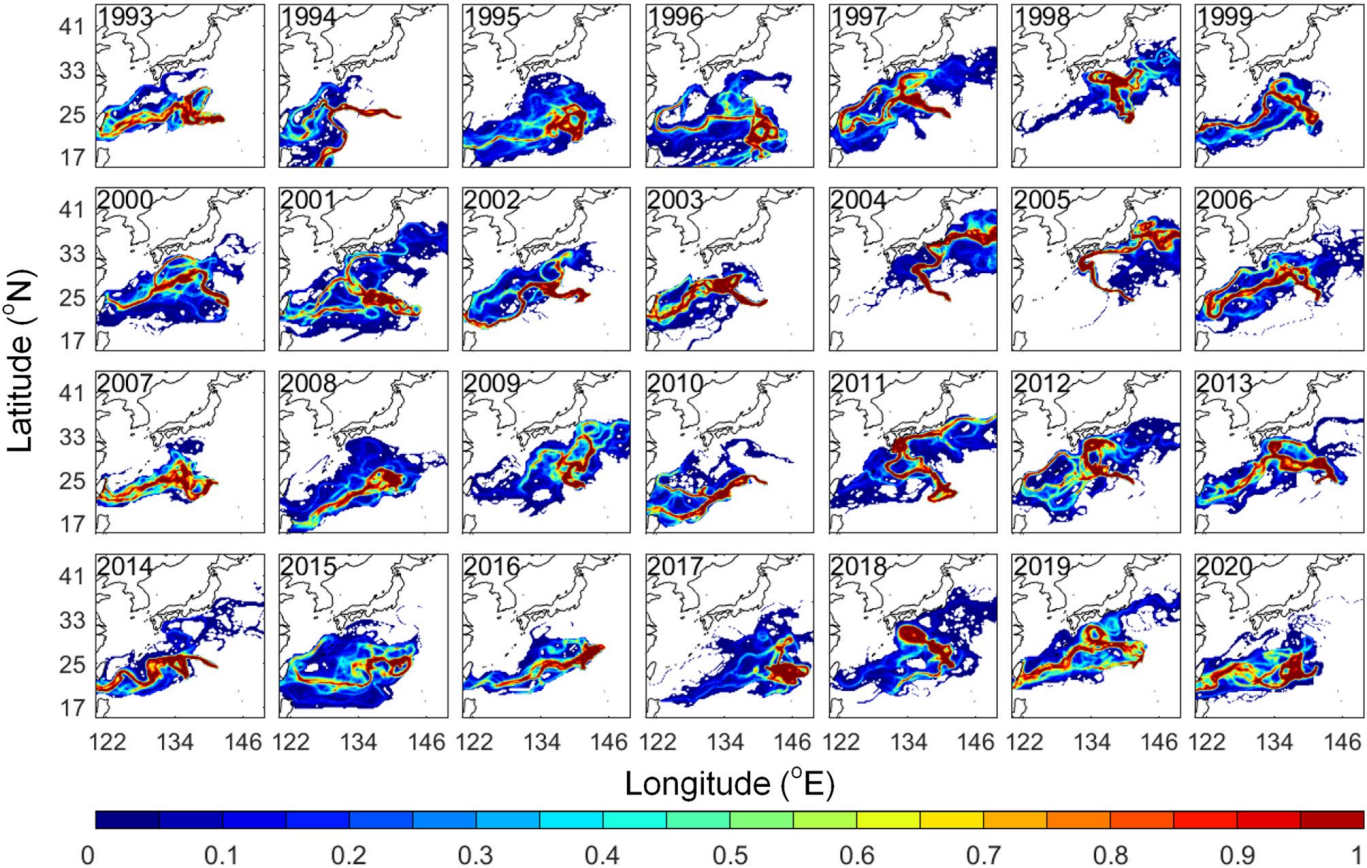
Normalized visitation frequency from 1993 to 2020 based on 2% windage. Figure is created using MATLAB R2011b (http://www.mathworks.com/).

**Figure 6:**
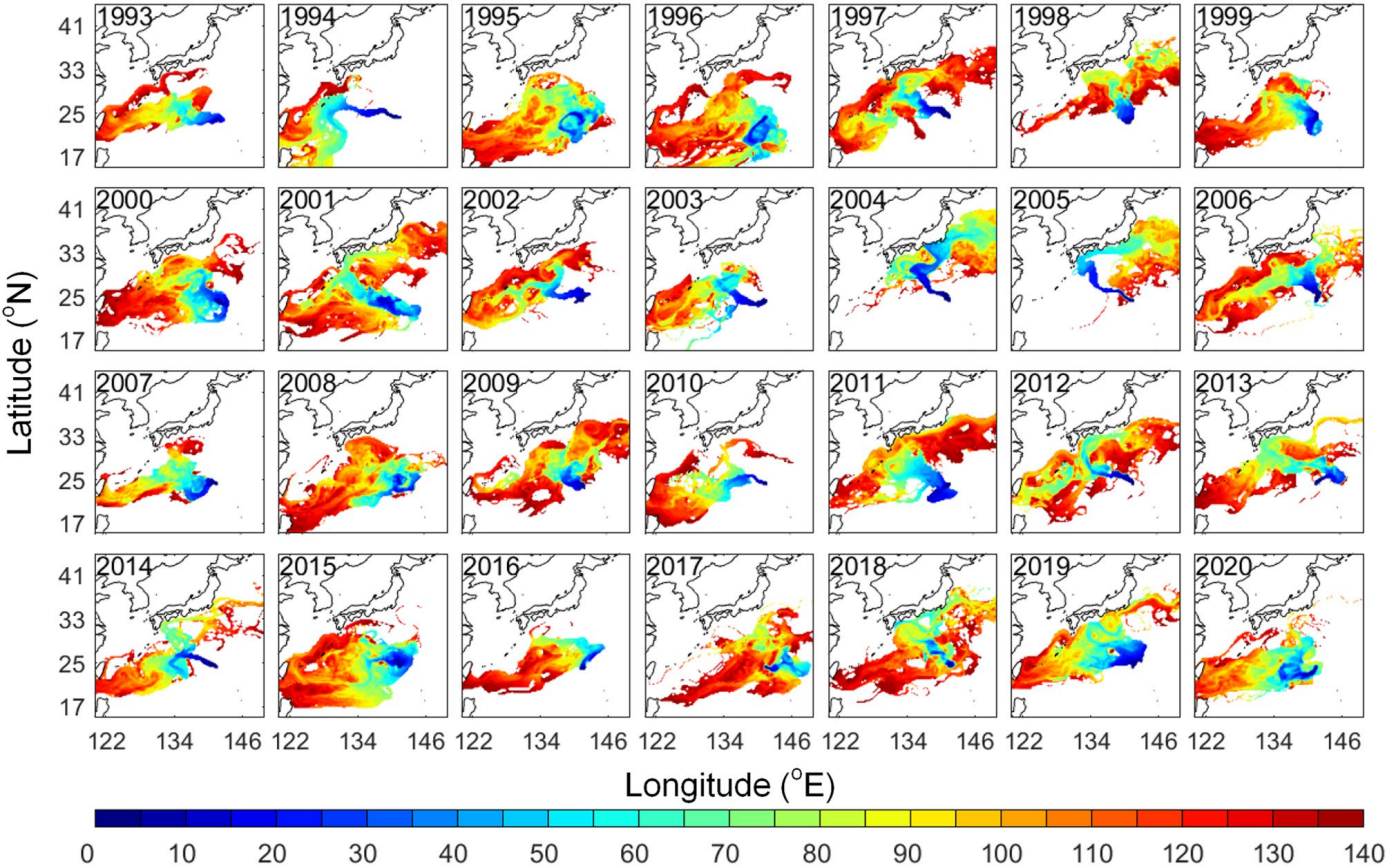
Time-evolving distribution (days) from 1993 to 2020 based on 2% windage. Figure is created using MATLAB R2011b (http://www.mathworks.com/).

**Figure 7:**
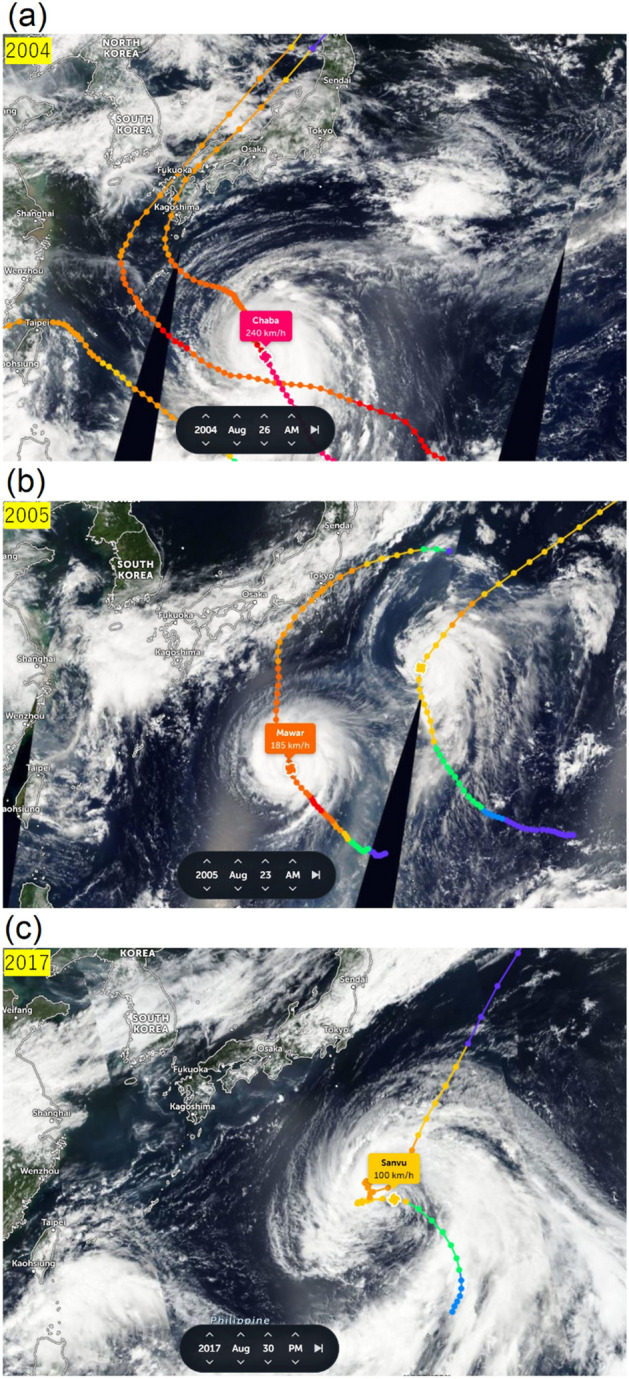
Satellite cloud images from zoom earth showing typhoons on (**a**) August 26, 2004 (**b**) August 23, 2005, and (c) August 30, 2017. Different colors represent different wind speeds, from blue (weak) to red (strong). Typhoon information and satellite image were observed from ZOOM EARTH (https://zoom.earth/).

The wind and ocean current conditions differed in the past 28 years, resulting in diverged distributions year by year in the 2% windage simulation (Fig. 5). The main dispersal routes (distinguished by greater visitation frequency) shown in most of the years were moving westward towards Okinawa and Taiwan. Interestingly, in 2004 and 2005 most virtual pumice moved northward directly toward Japan with only a few traveling westward towards Okinawa and Taiwan. Virtual pumice was confined in a narrow band and traveled towards Japan for the first 30 days in both years (Fig. 6). This northward movement in 2004 and 2005 was attributed to typhoons that occurred near the eruption site in late August (Fig. 7a, b). In another case in 2017, virtual pumice spread eastward and did not reach Taiwan and Japan until the end of the simulation, which was related to a tropical storm that occurred nearby (Fig. 7c). Although typhoon-affected time duration was short (~ few days), the extraordinarily strong winds (51 and 67 m/s in 2004 and 2005, respectively) could provide a non-negligible impact on pumice dispersion.

**Figure 8:**
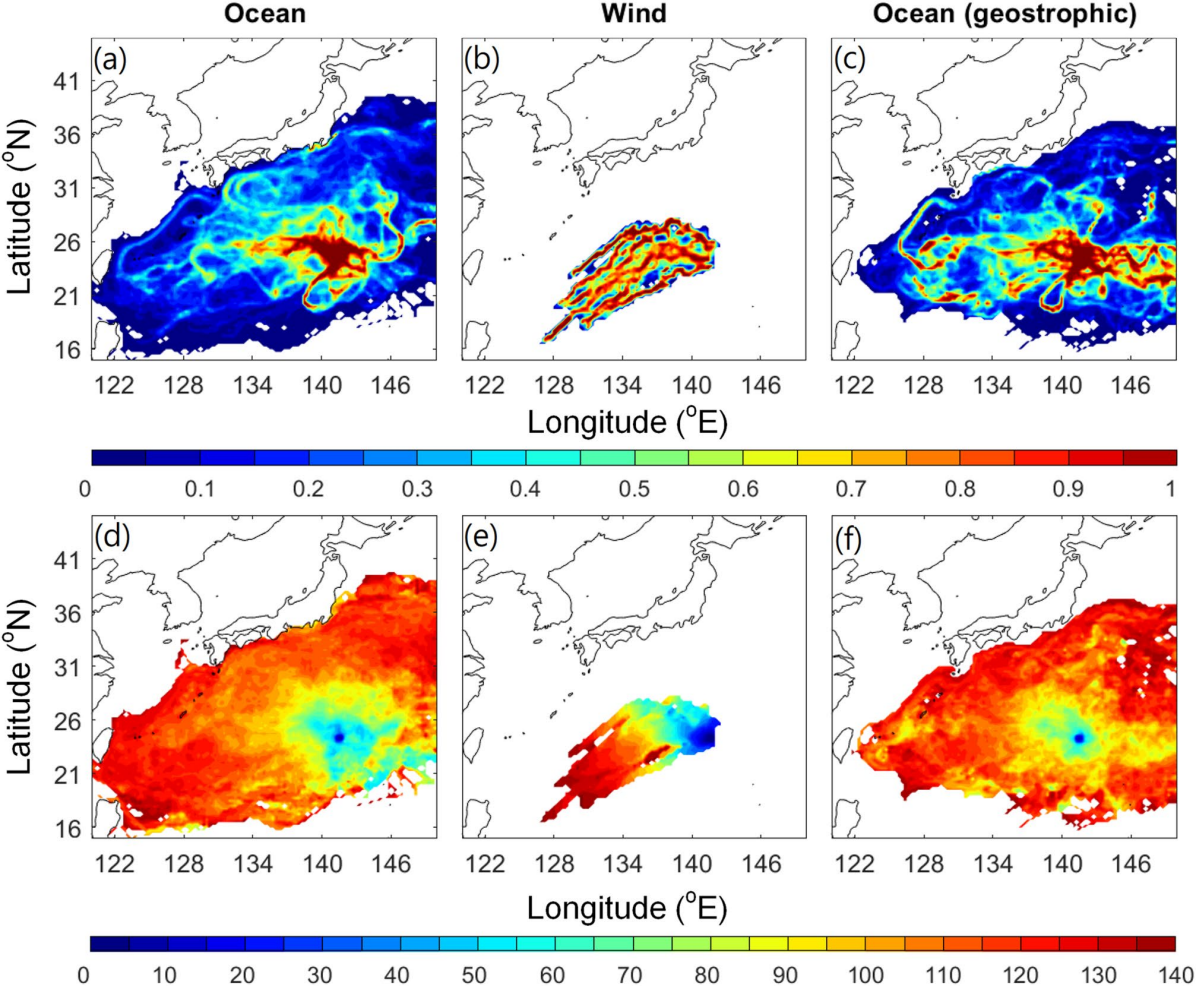
(**a**–**c**) Normalized visitation frequency and (**d**–**f**) time-evolving distribution (days) based on 28 years (1993–2020) daily hindcast with (a, d) 0% windage and ocean current; (**b**, **e**) 2% windage without ocean current; (**c**, **f**) 0% windage and geostrophic ocean current. Figure is created using MATLAB R2011b (http://www.mathworks.com/).

The rough calculation based on wind speed, ocean current, and satellite image described above seemed to suggest wind and ocean played a comparable role in pumice drifting. In order to distinguish the contribution of wind and ocean current in the present simulation, we conducted additional experiments with 0% windage (Fig. 8a, d, dominated by ocean current), without ocean current and with 2% windage (Fig. 8b, e, wind only), and 0% windage with geostrophic current (Fig. 8c, f, removing surface wind effect from ocean current). The experiment with 0% windage that considered the effect of ocean current showed a wide dispersion extending to Okinawa (Fig. 4a, 8a). The experiment with wind and without ocean current showed a less dispersed, southwestward drifting of around 1200 km in 4 months (Fig. 8b, e). In the 0% windage and geostrophic current case that eliminated the surface wind effect (i.e. northwestward Ekman drift induced by southwestward wind shown in Fig. 8b), the distribution was slightly less dispersed than the full ocean current (Fig. 8a, c), but remained much wider than wind effect only (Fig. 8b).

## Conclusions and discussion

The present study examined the long-term projection of pumice dispersal based on the reanalysis of the past 28 years of wind and ocean currents, considering the unavailable forecasts when a sudden event occurred. The composite of virtual pumice dispersion well captured the observed arrival to Okinawa in 2021. Multiple years of ensemble simulations included wide variability of wind and ocean current conditions, taking into account the model uncertainty. The multiple-year simulation may also serve as a reference for how pumice may propagate under different wind and ocean conditions, thus informing future projection.

**Figure 9:**
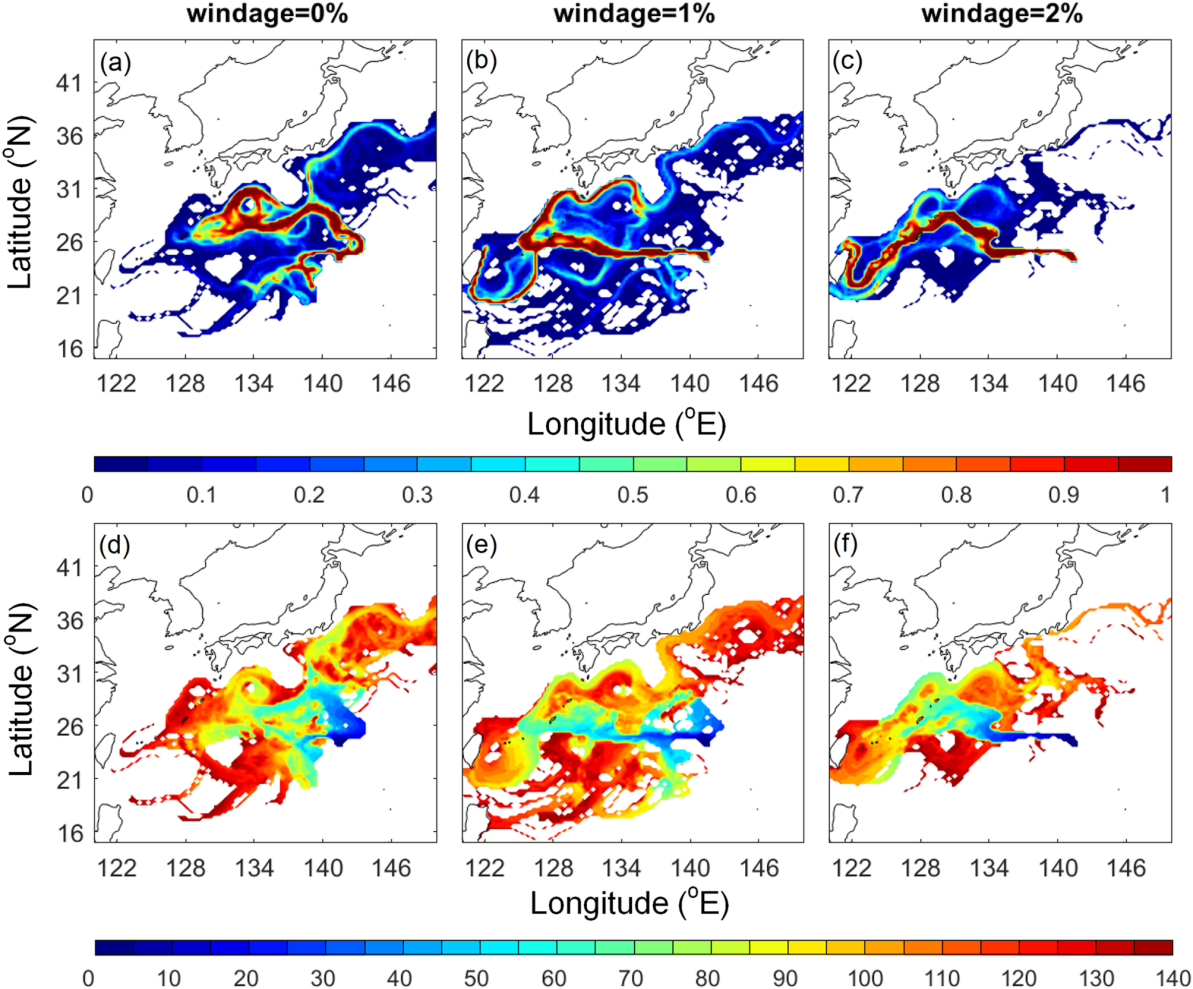
(**a–c**) Normalized visitation frequency and (**d**–**f**) time-evolving distribution (days) based on 2021 daily hindcast ocean currents with windage of (a, d) 0%, (b, e) 1%, and (c, f) 2%. Figure is created using MATLAB R2011b (http://www.mathworks.com/).

We repeated the simulation using the 2021 ocean current and wind data (now as a hindcast). The mean ocean current speed in 2021 between the eruption site and Okinawa was 0.094 m/s, slightly weaker than the past 28 years' average. The wind speed of 5.52 m/s however, was stronger than the ensemble mean. The estimated windage was about 2% to fit the observed arrival time to Okinawa. In the simulation, 1% and 2% windage both showed reasonable distribution propagation time compared to observation (Fig. 9). We should be aware that if the ocean currents and wind conditions exhibited considerable bias, the modelled dispersion from a single-year simulation could be very different to the observed dispersion. However, single-year simulation using hindcast/forecast data could still provide a reliable short-term prediction by frequently updating the initial condition based on satellite observation e.g. the simulation by the Application Laboratory of Japan Agency for Marine-Earth Science and Technology (https://www.youtube.com/playlist?list=PLKT1Tlr-tdGG85epmm6U9Gg7woBV4gd6p). In their simulation, they updated the initial pumice locations using the latest satellite observation provided by JAXA and did a short-term simulation based on the ocean forecasting system.

**Figure 10:**
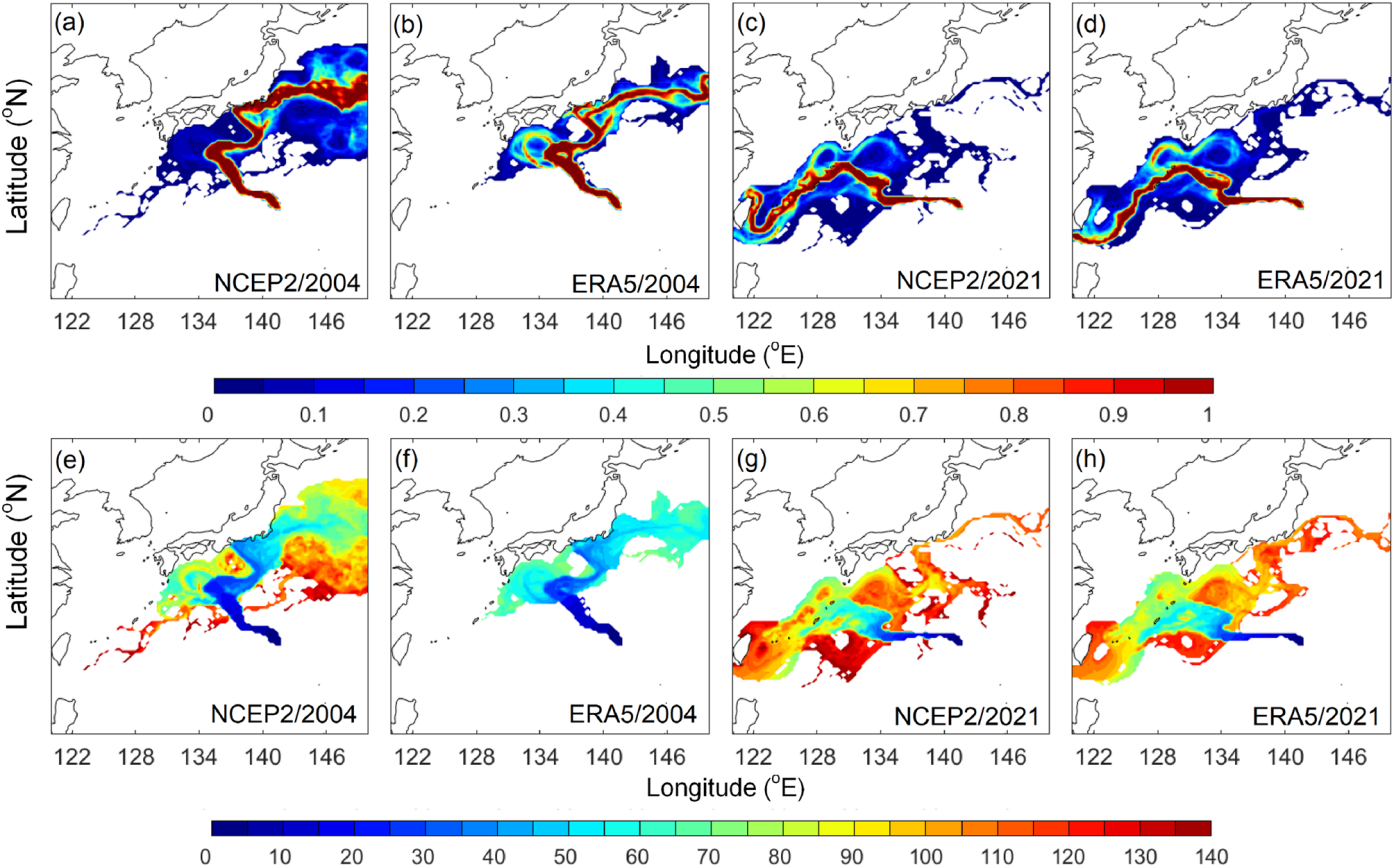
(**a**–**d**) Normalized visitation frequency and (**e**–**h**) time-evolving distribution (days) based on (**a**, **c**, **e**, **g**) NCEP2 and (**b**, **d**, **f**, **h**) ECMWF-ERA5 reanalysis wind with windage 2% for year (**a**, **b**, **e**, **f**) 2004 and (**c**, **d**, **g**, **h**) 2021. Figure is created using MATLAB R2011b (http://www.mathworks.com/).

We also considered whether coarse resolution of wind (i.e. NCEP reanalysis used in present study) or ocean products could significantly influence simulation results. We therefore repeated the 2% windage simulations for year 2004 (selected typhoon year) and 2021 (eruption year) using daily ECMWF-ERA5 reanalysis wind, which has higher horizontal resolution of 0.25 degrees. The general pumice distributions are similar between models using different wind products, yet there are some small-scale differences (Fig. 10). Because the wind rose map created with monthly ECMWF-ERA5 showed similar major wind directions between the eruption site and Okinawa (figure not shown), we expected a similar general distribution of ensemble projection using ECMWF-ERA5 wind, but the finer scale wind (i.e. near the coast) that were missing in the coarse resolution NCEP2 could make a difference to the distribution. These sensitivity experiments suggest small-scale features could be better represented by the higher resolution products. Overall, the ensemble projection method presented here and in previous study of marine oil spill^10^ worked well. We note that both events occurred in open ocean over time scales of a few weeks to months. If a future targeted event has a smaller spatial and temporal scale, then higher resolution input data may be required.

**Figure 11:**
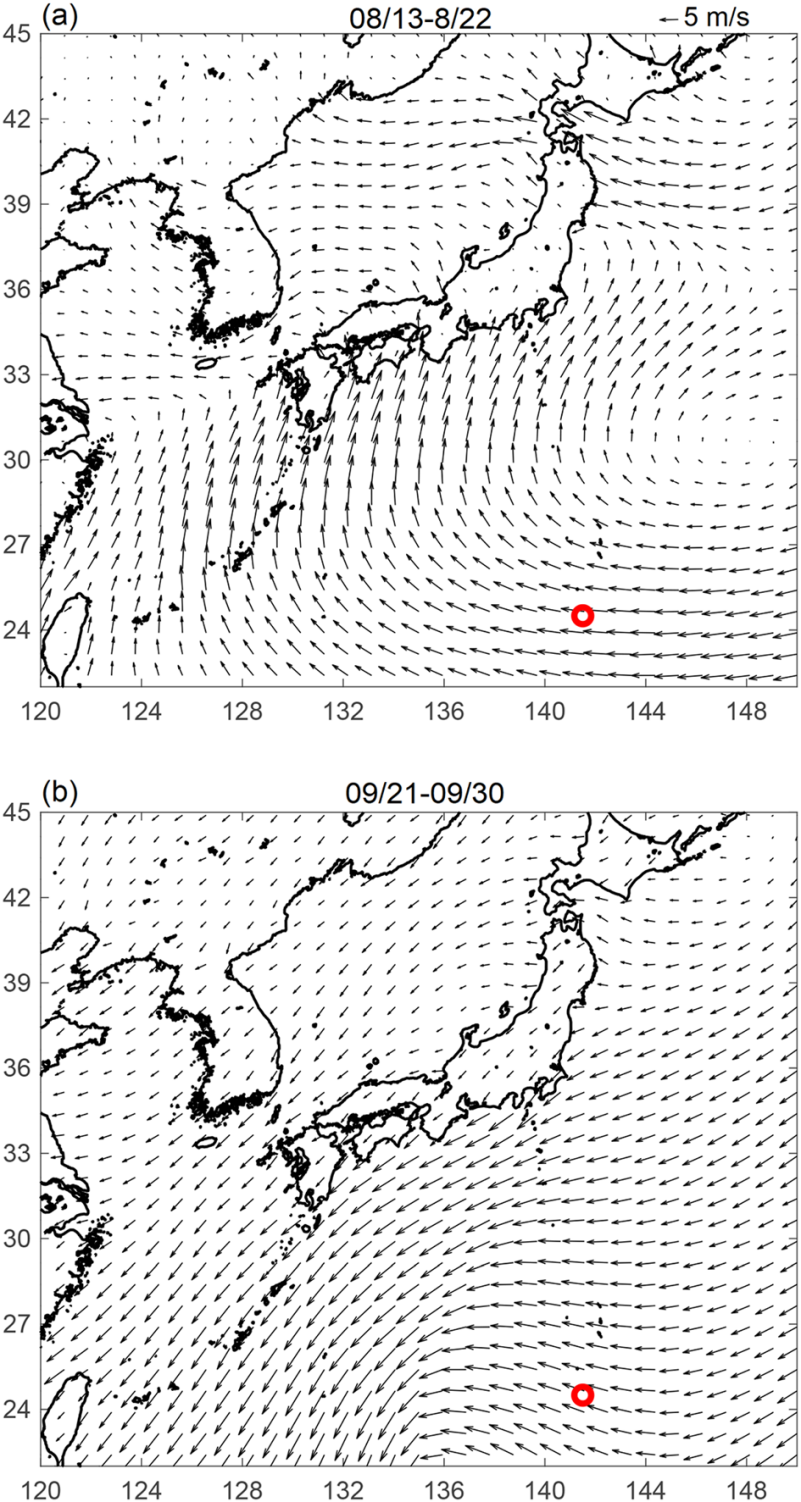
Average wind during (**a**) first 10 days after the eruption (13–22 August 2021), and (**b**) last 10 days of September (21–30 September 2021). Figure is created using MATLAB R2011b (http://www.mathworks.com/).

Apart from the prevailing wind and ocean currents, typhoons also played a role in affecting pumice dispersion. Typhoon occurrence near Fukutoku-Oka-no-Ba at the end of August is not unusual. In the past 28 years, 13 typhoons passed close to the eruption site during late August. Typhoons that occurred at the end of August seemed to have greater impact on the route of pumice dispersion than typhoons that formed in September or later. From the end of August to the end of the summer season, the prevailing wind in the western North Pacific was dominated by the subtropical high, and the wind near the eruption site was westward (Fig. 11a). If strong typhoon winds in August transported pumice to the north where the prevailing wind was blowing north, pumice would then continue to move towards the north instead of the original westward route. Conversely in September, when the prevailing wind changed directions with the change of season, the winds generally blow in the same direction from north to south near the eruption site (Fig. 11b). If pumice was disturbed by a September typhoon and shifted northward, it would thus still take a westward route. Indeed, although there was a typhoon that occurred at the end of September 2021, the virtual pumice—like the actual raft—did not make a rapid turn to Japan but instead still arrived in Okinawa and Taiwan (Fig. 9).

The Stokes drift induced by surface gravity waves could be a potential factor affecting pumice dispersion, although a previous study of the 2019 pumice raft from Volcano 0403-091 in Tonga found no apparent contribution from Stokes drift in their simulation^4^. The surface Stokes drift, assumed as a first-order approach to be acting in the direction of the wind^15^, had an annual mean value of 1–2 cm/s between Okinawa and Fukutoku-Oka-no-Ba^16^. Although Stokes drift was not included in our simulations, the better fit of the higher 2% windage model to the satellite-observed pumice dispersal may indeed reflect some contribution from Stokes drift because it contributes in the direction of the wind. If so, then separating the Stokes drift into an additional term would reduce the estimated windage by 0.2–0.4% (considering mean wind speed of 5.2 m/s in the study region). As noted by Jutzeler et al.^4^, the potential contribution of Stokes drift depends on the ability of pumice rafts to attenuate local wave heights and it may therefore become an important factor in dispersion of small (< 1 km^2^) and/or loosely-packed rafts. Accordingly, although our simulation did not directly include Stokes drift in the calculations, the high windage value (compared to previous studies) derived from our simulation may be due to a contribution of wind-forced Stokes drift that occurred as the initial compact raft gradually separated into more dilute pumice ribbons over time.

The ~ 0.2 m/s average travelling speed of the 2021 pumice raft estimated in based on satellite images (see Result section) is similar to average speeds calculated for other raft events e.g. ~ 0.2 m/s Volcano 0403-091, 2001^1^ ; ~ 0.23 m/s Home Reef, 2006^2^; ~ 0.02–0.19 m/s Havre, 2012^3^; and ~ 0.26 m/s Volcano 0403-091, 2019^4^. Notably, the pumice raft produced by the January 1986 eruption of Fukutoku-Oka-no-Ba, which also travelled to Okinawa, was slower (~ 0.1–0.18 m/s) and therefore took ~ 4 months to reach Okinawa^17^. This difference in travel time between the 1986 and 2021 rafts, and thus in the amount of time available in which to recognize the hazard, communicate warnings and undertake mitigation actions, highlights the need to better understand the controls on pumice dispersal. The pumice erupted in 1986 and 2021 appear very similar, with overlapping geochemical compositions and similar physical characteristics^17,18^, so it has been suggested (but not modelled) that the cause of their different average drift speeds could be due to variability in the Kuroshio counter-current in different seasons^18^.

However, the significant contribution of wind to dispersal speed shown in our study highlights that another potential cause is a difference in appropriate windage value for the 1986 and 2021 pumice. The relative importance of windage will vary according to how much (surface area and height) of the pumice is exposed above the waterline^3^, which will differ according to the shape, porosity (i.e. vol% bubbles), and degree of waterlogging (i.e., filling of bubbles within the rock by infiltrating seawater that increases overall density^19^) of individual clasts. Pumice size and shape can vary greatly depending upon eruption characteristics and can be up to meters in size^20,21^, although repeated collisions within the raft will tend to fragment this fragile vesicular material into smaller pieces over time. Even among pumice with the same size, shape and porosity there can be considerable variability in bubble characteristics (e.g. size, connectivity, size of connecting pore throats) that control how rapidly pumice becomes waterlogged^22^; differences in eruption depth, eruption style and timescales of pumice cooling within the water column versus in a subaerial plume also affect how much buoyancy is lost due to water ingestion before reaching the sea surface^23–25^. Physical characteristics and eruption conditions could therefore combine in complex ways to ultimately define pumice size and buoyancy, hence windage. Observations of the 2021 Fukutoku-Oka-no-Ba eruption indicate that the raft pumice was rapidly delivered to the sea surface without significant cooling or waterlogging due to the high magma discharge rate of the eruption^6^. By contrast, the maximum known plume height of the (admittedly less well observed) 1986 eruption was only 4 km^26^, which may indicate a less vigorous eruption style in 1986. While the geochemical and physical characteristics of the 1986 and 2021 pumice overlap, we therefore note that different eruption processes (e.g., a slower ascent and/or less insulation from surrounding seawater within a less powerful plume) may have affected the degree of water ingestion during raft formation. As well as seasonal variation in ocean currents and winds, eruption-related differences in pumice windage should therefore also be considered as a potential factor in the slower dispersal of the 1986 pumice relative to the 2021 pumice.

Increasingly high spatial and temporal resolution of satellite observations will enable further testing and refinement of dispersal models during future pumice raft events, including the importance or otherwise of accounting for Stokes drift and other effects such as tides and waves. Given the current evidence regarding the variability and importance of windage values for accurate dispersal forecasting, we propose that research to constrain how pumice windage varies with bubble characteristics and waterlogging processes is also an important step to undertake in advance of the next raft eruption.

## Data Availability

The ocean reanalysis data used in this study is available in APRDC database (http://apdrc.soest.hawaii.edu/datadoc/fra-jcope2.php). The wind reanalysis from NCEPII can be downloaded from https://psl.noaa.gov/data/gridded/data.ncep.reanalysis2.html. Satellite observed pumice locations can be downloaded from (https://earth.jaxa.jp/karuishi/).
